# Editorial: Regulation of plant organelle biogenesis and trafficking

**DOI:** 10.3389/fpls.2023.1211807

**Published:** 2023-05-26

**Authors:** Jean-Marc Neuhaus, Peter Pimpl, Qiong Zhao, Hao Wang

**Affiliations:** ^1^ Laboratory of Cell and Molecular Biology, University of Neuchâtel, Neuchâtel, Switzerland; ^2^ Key Laboratory of Molecular Design for Plant Cell Factory of Guangdong Higher Education Institutes, Institute of Plant and Food Science, Department of Biology, School of Life Sciences, Southern University of Science and Technology, Shenzhen, China; ^3^ School of Life Sciences, East China Normal University, Shanghai, China; ^4^ Department of Cell and Developmental Biology, College of Life Sciences, South China Agricultural University, Guangzhou, China

**Keywords:** plant organelle, organelle biogenesis and trafficking, SNARE, Golgi structure, oil body (OB), mitogenome, vesicle traffic

The emergence of membrane-bounded organelles is a hallmark of eukaryotic cells, allowing multiple and incompatible biochemical processes to occur simultaneously (Gomes and Shorter, 2019; Mathur, 2020). Each organelle contains a specific set of proteins, lipids, and cofactors that define its characteristic morphology and function. Meanwhile, the activities and functions of cellular organelles must be well orchestrated for the cell to function properly as a biological unit (Cohen et al., 2018; Perico and Sparkes, 2018). The process of organelle biogenesis involves the coordinated expression of genes, signaling pathways, and molecular interactions that result in the formation and maintenance of organelle structure and function. Furthermore, proper regulations of organelle subcellular localization, trafficking, and interactions are crucial for cell growth, development, and homeostasis (Lippincott-Schwartz et al., 2000; Noack and Jaillais, 2017). Thus, organelle biogenesis, function, and diversity are fundamental and critical to every cell.

The organelles play essential roles in various physiological and metabolic processes, including photosynthesis, cell wall construction, hormonal distribution, and cell signaling (Saftig and Klumperman, 2009; Kang et al., 2011; Perico and Sparkes, 2018; Li et al., 2020; Robinson, 2020; Shimizu et al., 2021). Moreover, the trafficking and interactions of organelles within cells are dynamic processes that involve coordinated action of various components, including motor proteins, cytoskeletal elements, and membrane trafficking regulators such as small GTPases and soluble N-ethylmaleimide-sensitive-factor attachment protein receptors (SNAREs). They are also influenced by environmental cues such as light, temperature, and various other biotic and abiotic stresses (Uemura and Ueda, 2014; Luo et al., 2017; Yun and Kwon, 2017; Rosquete and Drakakaki, 2018; Won and Kim, 2020). Understanding the mechanisms underlying plant organelle biogenesis and trafficking is essential for improving plant growth and development, as well as for devising new biotechnological applications that rely on the targeted delivery of molecules to specific plant cells or tissues (Miao et al., 2008; Michoux et al., 2011; Ou et al., 2014; Lomonossoff and D’Aoust, 2016).

In this Research Topic issue, several essential aspects of organelle biogenesis, membrane trafficking, and protein sorting in both model and non-model plants have been advanced. In eukaryotic cells, new proteins and lipids are delivered from their site of synthesis in the endoplasmic reticulum (ER) to various subcellular destinations. The Golgi apparatus is a central organelle in secretory membrane traffic and sorting. In plant cells, the Golgi additionally serves as a major biosynthetic organelle for synthesizing polysaccharides, which are key elements for the plant cell wall construction (Reyes et al., 2011; Chung and Zeng, 2017). The morphogenesis and maintenance of the stacked cisternal structure of the Golgi body are critical for its biological functions. Rui et al. provide an update on key regulators that mediate ER-Golgi, *intra*-Golgi, and *post*-Golgi trafficking pathways. Furthermore, they focus on functional molecules that participate in retrograde vesicular transport from *trans*-Golgi to *cis*-Golgi cisternae, including Arf1, coatomer, the COG complex, SYP31 and 32, Rab GTPases and Golgi matrix proteins.

In addition, soluble N-ethylmaleimide-sensitive factor attachment protein receptor (SNARE) proteins are a family of proteins that are essential to mediate membrane fusion in eukaryotic cells. They also play a crucial role in various cellular processes, including organelle interactions, vesicular trafficking, cytokinesis, and are involved in growth, development and stress responses in plants (Grefen and Blatt, 2008; Kwon et al., 2020). VPS45 is a protein that belongs to the Sec1/Munc18 family and interacts with and regulates Qa-SNARE function during membrane fusion. Mugume et al.identified a mutant of VPS45, which is caused by a point mutation in the *VPS45* gene that differs from the lethal *vps45* knockout mutation in Arabidopsis. They further revealed that impaired VPS45 function causes vacuolar defects and leads to a loss of turgor pressure that is needed for proper tip growth. Besides, Luo et al. summarized recent progress in understanding the biological functions and signaling network of SNAREs in vesicle trafficking and the regulation of root growth and development in Arabidopsis.

Proteins of the secretory pathway are transported from the Golgi stack to the *trans*-Golgi network (TGN) for sorting and trafficking to different subcellular localizations. In plant cells, the TGN has been identified as an independent organelle that also functions like the early endosome (EE) of animal cells and is therefore also referred to as TGN/EE (Dettmer et al., 2006; Lam et al., 2007; Richter et al., 2009; McKay et al., 2022). It is also the source for the biogenesis of the multivesicular bodies (MVBs), the late endosomes (LEs) that facilitate the trafficking to the lytic vacuole (Scheuring et al., 2011). Shimizu and Uemura review the recent results from fast live imaging by spinning disk confocal microscopy and from 3D reconstructions by electron tomography that allowed to distinguish the Golgi-associated TGN (GA-TGN) from the Golgi-independent TGN (GI-TGN) and also specialized domains within the GA-TGN. The markers AP-1, Epsin1, clathrin and VAMP721 are associated with a domain involved in trafficking to the plasma membrane, while AP-4, MTV1 and VAMP727 are associated with another domain involved in trafficking to the vacuole. Whether clathrin is involved in the latter pathway is unclear. The GI-TGN derives from the former domain of the GA-TGN and produces AP-1/clathrin-coated vesicles, which may play a role in retrograde trafficking. The secretory trafficking is further complicated by the separate sorting and transport of proteins to different domains of the plasma membrane, as is seen in root endodermal cells. Inhibitors differently affect transport to the two target membranes, but this sorting has not yet been localized within the TGN. Finally, not all protein trafficking to the vacuoles may implicate the TGN, as AP-3 mediated sorting of several proteins may well already occur at the *trans*-Golgi.

Plant adaptation relies on neofunctionalization of the endomembrane system (ES) to acquire new organelles, which may serve for secondary metabolism. This approach is often ignored due to the intricacy of angiosperms. Bryophytes, with their simple cellular structures and unique organelles such as oil bodies (OBs), are great models for studying the role of the endomembrane system (ES) in the production of plant secondary metabolites (PSMs), as well as how new organelles are acquired during evolution (He et al., 2013; Kanazawa et al., 2020; Romani et al., 2020; Romani et al., 2022). Liverwort’s OBs are single-membrane organelles containing lipophilic globules and PSMs in a protein matrix, and are typically found in gametophyte cells (He et al., 2013; Romani et al., 2022). Recent studies on OBs in *Marchantia polymorpha* have identified several key transcriptional factors, including ERF, MYB and HDZ, which coordinate the redirection of the secretory pathway toward OB formation (Romani et al., 2020). Research on the *M. polymorpha* SNARE protein (MpSYP12B) found in OBs suggests that these organelles may have originated from the expansion of secretory trafficking systems in plants (Kanazawa et al., 2020). Furthermore, the same research group recently identified that maintaining the shape of the OB is a complex process that involves the COPI components (Kanazawa et al.). Systemic research on OB provides compelling evidence to support the notion that the redirection of the secretory pathway contributes to OB formation and shape, although the physiological significance of maintaining OB shape requires further study (Kanazawa et al., 2020). This research underscores the importance of studying OBs in bryophytes and highlights the need for similar future studies on non-model organisms to maximize our understanding of organelles and trafficking. Ultimately, such studies can enrich our knowledge in this field.

Mitochondria, the cell’s “powerhouse”, produce respiratory ATP and are essential for eukaryotic life. Most mitochondrial proteins are encoded by the nuclear genome, synthesized in the cytosol, and translocated to the mitochondria (Møller et al., 2021). Nevertheless, mitochondria are known as semi-independent organelles, which also contain their own mitochondrial genome (mitogenome) (Barrera-Paez and Moraes, 2022). Understanding the plant mitogenome can help us better understand the function of mitochondria in plant cells, and develop strategies to improve plant health and crop yields by editing the mitochondrial genome (Yang et al., 2022). Mitogenomes from different plant species can be used to study the evolution of plant lineages, as well as to investigate the relationships between plant species and other organisms such as fungi and bacteria. In addition, plant mitogenomes can also serve as a tool for studying the phylogenetics of different plant species (Zardoya, 2020). In recent years, high-throughput sequencing techniques have accelerated the sequencing of mitogenomes and uncovered the great diversity of organizations, gene contents, and modes of replication and transcription found in living eukaryotes. Feng et al. have assembled complete mitogenomes of 23 species that cover seven families of Fagales. By their analysis of their mitogenomic structures and capacity in phylogeny, they offer a fresh perspective on the evolution of mitochondrial genomes and the variations in their size. Furthermore, Bi et al. completed the assembly of the complete mitochondrial genome of *Populus simonii* and provide insights into the stability of genome sizes and gene contents in the genus *Populus*.

Together, the biogenesis and trafficking of plant organelles involve complex cellular processes that are coordinated by various signaling pathways and molecular interactions. Recent advances in our understanding of the regulation of plant organelle biogenesis and trafficking have provided new insights into the complex cellular processes underlying plant growth and development. Further research in this fascinating and challenging area will undoubtedly uncover new regulatory pathways and molecular mechanisms, providing new targets for plant/crop improvement, environmental sustainability, and biotechnological applications.

## Author contributions

All authors listed have made a substantial, direct, and intellectual contribution to the work and approved it for publication.
